# Hepatocellular Carcinoma in a 24-Year-Old Female with Beckwith–Wiedemann Syndrome: A Case Report and Review of the Literature

**DOI:** 10.1155/2020/8811296

**Published:** 2020-10-07

**Authors:** Carolyn G. Ahlers, Quoc-Huy Trinh, Martin Montenovo

**Affiliations:** ^1^Vanderbilt University School of Medicine, 1161 21^st^ Ave S, Nashville, TN 37232, USA; ^2^Vanderbilt University Medical Center Department of Pathology, Microbiology, and Immunology, 1211 Medical Center Dr, Nashville, TN 37232, USA; ^3^Vanderbilt University Medical Center Division of Hepatobiliary Surgery and Liver Transplantation, 1211 Medical Center Dr, Nashville, TN 37232, USA

## Abstract

In this report, the case of a 24-year-old female with Beckwith–Wiedemann Syndrome (BWS) who was diagnosed with well-differentiated hepatocellular carcinoma (HCC) is described. While BWS has been associated with childhood embryonal tumors, most commonly Wilms tumors and hepatoblastomas, this is the first case report to describe HCC in an adult with BWS. Although HCC typically occurs in elderly adults or those with underlying liver disease, in this case, we show that HCC can occur in a young adult with BWS without any underlying liver disease.

## 1. Introduction

Beckwith–Wiedemann Syndrome (BWS) is a rare genetic disorder characterized by macroglossia, abdominal wall defects, facial nevus flammeus, hypoglycemia, organomegaly, and hemihypertrophy. The syndrome is also associated with many childhood embryonal malignancies, most frequently Wilms tumors and hepatoblastomas. In this case report, we describe the diagnosis of hepatocellular carcinoma (HCC) in a 24-year-old woman with BWS. This is the first case report to describe HCC in an adult with this syndrome.

## 2. Case Report

A 24-year-old Caucasian woman with a past medical history significant for congenital BWS presented with vague abdominal pain that waxed and waned in intensity beginning in August 2018. The patient had clinical manifestations of BWS at birth with characteristic umbilical hernia, macroglossia, and hemihypertrophy of her right lower extremity. Genetic analysis confirmed the diagnosis of BWS in January 2011 with uniparental disomy of the chromosome 11 region, hypermethylation of the H19DMR (IC1) loci, and hypomethylation of the KvDMR (IC2) loci. In addition to diagnosis of BWS, the patient's past medical history was significant for partial pancreatectomy due to pancreatic cystic lesions, left mastectomy, and several right-sided lumpectomies due to breast fibroadenomas, refractory immune thrombocytopenic purpura requiring splenectomy, and systemic lupus erythematosus.

In May 2019, she was found on CT chest, abdomen, and pelvis to have interval development of parenchymal heterogeneity and multiple enhancing nodules and masses in the liver measuring up to 3.6 cm in the right dome (Video 1). Two months later on MRI, the patient was found to have a 3 cm lesion in the right hepatic dome of the liver (Video 2). Tumor and autoimmune laboratory markers at this time demonstrated a normal alpha fetoprotein at 2.7, a mildly elevated carcinoembryonic antigen at 3.8, an elevated carbohydrate antigen 19–9 at 136, a positive antinuclear antibody 1:1280, and a positive smooth muscle (*F*-actin) immunoglobin G. Ceruloplasmin, antimitochondrial antibody, alpha-1-antitrypsin, and viral hepatitis serologies were negative. Liver function was preserved, but a marker of liver inflammation, aspartate aminotransferase, was mildly elevated to 65. Alanine aminotransferase was normal at 39, and the patient's alkaline phosphatase was elevated to 211.

**Figure 1:**
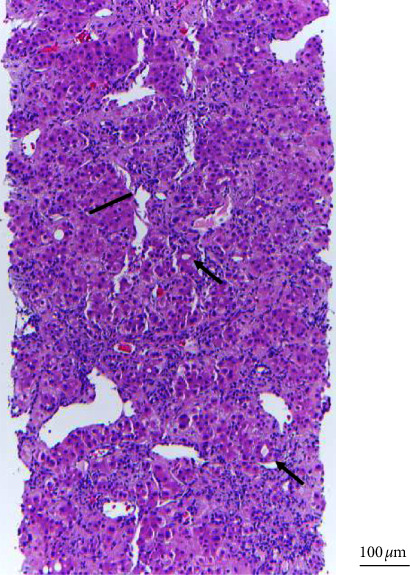
Pathology of the ultrasound-guided liver biopsy showed hepatocellular proliferation with thickened cell plates and pseudoglands.

Given the above imaging and laboratory results, an ultrasound-guided liver biopsy was performed by interventional radiology in October 2019. The biopsy was described as a hepatocellular proliferation with thickened cell plates and pseudoglands (Figure 1). No portal tracts were observed, but some unpaired arteries were noted. A low-grade, well-differentiated hepatocellular neoplasm was diagnosed due to the frequent pseudoglandular formations and the thickened cell plates. At this time, the patient denied other symptoms, including fever, chills, nausea, vomiting, weight loss, headache, dyspnea, flank pain, constipation, diarrhea, melena, hematochezia, focal weakness, or back pain. Right posterior sectionectomy of the liver was performed in December 2019.

**Figure 2:**
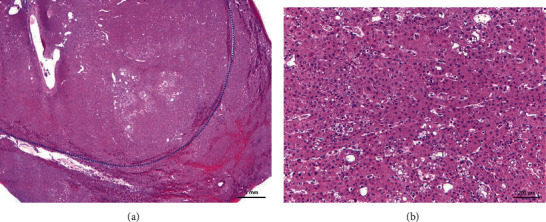
Pathology of the surgical specimen showed diffusively thickened cell plates and pseudoglands (a), (b).

**Figure 3:**
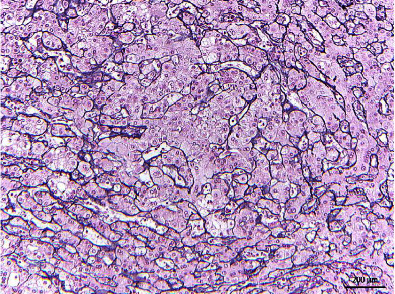
A reticulin stain confirmed the presence of thickened cell plates in the surgical specimen.

Serial sections of the surgical specimen revealed a 2.9 × 2.8 × 2.0 cm tan, soft, and hemorrhagic mass with irregular borders. Histological examination demonstrated similar findings to those seen in the biopsy, with diffusely thickened cell plates and pseudoglands (Figures 2(a) and 2(b)). The reticulin stain confirmed the presence of thickened cell plates (Figure 3). The tumor was extensively sampled, and no areas were concerning for embryonal or fetal differentiation, which would have been suggestive of hepatoblastoma. The background of the liver showed mass-effect changes and focal perisinusoidal fibrosis. The surgical specimen confirmed the diagnosis of low-grade, well-differentiated hepatocellular carcinoma. The patient was discharged home a week later. Written informed consent was obtained from the patient for this case report.

## 3. Discussion

To our knowledge, this is the first case report in the literature to describe hepatocellular carcinoma (HCC) in an adult with BWS. BWS is a rare genetic syndrome which may present with macroglossia, abdominal wall defects, facial nevus flammeus, hypoglycemia, organomegaly, and hemihypertrophy. [1] The prevalence of BWS is one per 10,340 live births. [2] The disease is usually due to genetic or epigenetic changes affecting the 11p15.5 chromosome region. [1] A molecular defect affecting imprinted genes in the 11p15 chromosome region is responsible for approximately 80% of BWS cases, with loss of methylation at the maternal IC2 allele occurring in approximately 50% of patients and gain of methylation at the IC1 allele occurring in 5–10% of patients. Segmental paternal uniparental disomy is responsible for approximately 20% of disease cases. CDKN1C mutations are responsible for 5% of sporadic cases of BWS, and in 20% of BWS cases, a molecular diagnosis cannot be reached [1]. The patient in this case report had hypomethylation of IC2, hypermethylation of IC1, and segmental paternal uniparental disomy on genetic analysis.

BWS is associated with many childhood benign and malignant malignancies, most commonly embryonal carcinomas. The overall estimated neoplastic risk is 8%. [3] The most common tumors associated with BWS are Wilms tumors and hepatoblastomas, though rhabdomyosarcomas, adrenal cortical carcinomas, mesenchymal liver hamartomas, and neuroblastomas have been reported. [4, 5] Certain genetic mutations of BWS are associated with a higher oncologic risk than others. Uniparental disomy is associated with an overall neoplastic risk of 16%, while IC1 gain of methylation is associated with an overall risk of 28.1%. IC2 loss of methylation is associated with an overall risk of 2.6%. [1] Overall, the oncologic risk is highest for patients with BWS in the first two years of life, [1] and after ten years of age, the tumor risk approaches that of the general population. [6] There is no evidence of an increased risk of malignant tumors in adults with BWS, [1] although hepatoblastoma, acute lymphoblastic leukemia, adrenal adenoma, testicular Sertoli cell tumor, and three benign tumors (hepatic hemangioma, uterine myoma, and mammary fibroepithelioma) have been reported in young adults with BWS [7].

There have been no case reports in the literature to describe HCC in an adult with BWS. HCC is the most common primary hepatic malignancy. [8] The majority of hepatocellular carcinomas occur in patients with underlying liver disease. [9] Risk factors for HCC include chronic alcohol consumption, chronic hepatitis B or C infection, hereditary hemochromatosis, aspergillus-derived aflatoxin exposure, alpha-1-antitrypsin deficiency, Wilson disease, glycogen storage disease, diabetes, obesity, and nonalcoholic fatty liver disease. [8] Increased age is also a risk factor for HCC development, with latest estimates in the United States suggesting that the incidence of HCC peaks above the age of 70 years. [10–12] According to the International Agency for Research on Cancer, the age-standardized incidence rate of liver cancer in people 20–34 years old worldwide is 0.84 per 100,000. [13] Using this incidence rate, the probability of a 24-year-old having HCC and BWS (incidence of 1:10,340) due to uniparental disomy (approximately 20% of BWS cases) is 2 × 10^−10^. This indicates that HCC in a young adult with BWS is unlikely to be due to chance.

This is the first case report in the literature to describe HCC in a young adult with BWS. While the risk for malignancy is highest for patients with BWS during the first decade of life, this case report describes the case of HCC in a 24-year-old woman with this syndrome, highlighting the need for life-long cancer surveillance in this patient population.

## Data Availability

The raw data used to support the findings of this study are included within the article and the supplementary information file.

## Supplementary Materials

Supplementary Materials Video 1: CT chest, abdomen, and pelvis showed interval development of parenchymal heterogeneity and multiple enhancing nodules and masses in the liver measuring up to 3.6 cm in the right dome. Video 2: On MRI, the patient was found to have a 3 cm lesion in the right hepatic dome of the liver.
